# Bibliography

**Published:** 1903-06

**Authors:** 


### Bibliography.

THE INTERNAL SECRETIONS AND THE PRINCIPLES OF MEDICINE. By Charles E. de M. Sajous, M.D., Fellow of the College of Physicians of Philadelphia; formerly Lecturer on Laryngology and Dean of the Faculty in the Medico-Chirurgical College, etc. Volume First. With forty-two illustrations. F. A. Davis Company, Publishers, Philadelphia, 1903.

In the April issue of this journal notice was given of the publication of this book, and a portion of the "Preface and Summary of Contents" was given in the language of the author, to afford some idea of the contents of the then unpublished work. At an earlier period than was then anticipated this remarkable book is furnished the reading medical public.

When Dr. Sajous announced the conclusions at which he had arrived regarding the ductless glands, it aroused great interest in medical and lay circles, and the promised book was eagerly anticipated as possibly confirming the assertions of the author. Whether these hopes are sustained must be left to those most competent to decide the question.

There can be no doubt, however, of the fact, apparent throughout the volume, that the author has made an exhaustive examination of the medical literature of the world to sustain his views. In the opinion of the reviewer, Dr. Sajous has in the past done more in this direction than any other individual in the medical ranks. This book, therefore, must be regarded as a mine of information regarding this special subject, whatever other and antagonistic views be entertained. The pages bristle with quotations; indeed, these are bewildering at times, and in some degree tend to obscure the author's original ideas.

This first volume covers eight hundred pages, and the author says of it, "We have always deemed it our duty to closely follow the development of the various branches of medical science as the yearly panorama passed before our eyes, in the hope that we might eventually collate the necessary elements for a more solid foundation than medicine now possesses."

He further informs the readers why the main subject of this book was early called to his attention, for he says, "Among the subjects which had received attention during our preliminary inquiry was the physiology of the ductless glands. Although the thyroid body had been studied by a larger number of investigators than the adrenals, the latter seemed to us to present a feature directly connected with the problem; *i.e.*, the marked affinity of adrenal extractives for oxygen. We therefore determined to follow the clue this afforded as far as recorded facts would permit, and to trace its connections beyond the field of physiology if possible."

He again states: "It thus became evident that the red corpuscles were not the only carriers of oxygen, and that the blood-plasma played an important part in the distribution of this gas. Indeed, we subsequently ascertained that the red corpuscles were secondary factors in this important function,—*i.e.*, mere carriers, pack-mules, as it were,—and that it was the oxygen-laden adrenal secretion dissolved in the plasma itself which carried on all the oxidation processes of the organism."

That the author has gathered his ideas from the world's medical literature is demonstrated by his admissions in the following paragraph: "We fully realize, however, that our factors were necessarily drawn from a mere fraction of existing literature,—though a vast amount of the latter had to be scrutinized,—and that the balance of recorded data and future work of the galaxy of brilliant workers which our profession contains in all lands may eventually completely transform our views. . . . Yet, if our aim is properly interpreted, it will become apparent that we have encompassed the whole field of medical science in our labors, in the hope that a broad horizon would enable us to discover its weaker parts."

It will thus be seen that the author's deductions are mainly founded upon the original labors of widely scattered investigators. While this is true, no one can complain that the author has taken undue advantage of these accumulated facts to build up an unwarranted hypothesis; on the contrary, he constantly desires the facts presented to speak for themselves.

In regard to the action of poisons upon the adrenals the author has this to say: "It is evident that the adrenals are submitted to excessive activity when toxics are introduced into the organism, and that the local lesions are the expression of a physiological function utilized beyond its normal limits."

This in regard to the action of uric acid has some special interest to dentists: "*Uric acid, notwithstanding the prevailing belief to the contrary, is chemically harmless.* It has been fed to animals or injected into their blood in very large doses without giving rise to untoward symptoms. Even its continued administration has failed to bring about the least pathological change in the structures which it is generally thought to assail, as shown experimentally by A. C. Crofton."

In summing up the conclusions at which the author arrived in regard to the "Pituitary, Thyroid, and Adrenals as a System," he states:

"1. The thyroid gland supplies the blood with a secretion which has for its object to sustain the functional activity of the anterior pituitary body.

"2. The anterior pituitary body is directly connected with the adrenals through the cervico-thoracic ganglia, the splanchnic nerves, and the semilunar ganglia of the sympathetic nervous system.

"3. The thyroid gland, the anterior pituitary body, and the adrenals are functionally interdependent, and constitute a system through which cardiac action, respiration, and general cellular oxidation are maintained.

"4. The thyroid gland sustains the normal functional activity of the anterior pituitary body, while the latter in turn maintains the normal activity of the adrenals.

"5. The functional activity of the anterior pituitary body is increased when the blood contains an excess of thyroid secretion or sufficiently active toxics—bacterial toxins, poisons, physiological toxalbumins, etc.—to compromise the general cellular integrity of the organism.

"6. The functional activity of the adrenals is increased proportionally with that of the anterior pituitary body when the latter's activity is increased from any cause.

"7. The functional activity of the anterior and posterior pituitary bodies is passively decreased when the blood contains an insufficient proportion of thyroid secretion or is inadequately oxygenated, or when from any cause its intrinsic metabolism is reduced or impaired through the deficiency of any of its molecular constituents.

"8. The functional activity of the anterior pituitary body is actively decreased when the blood contains a sufficiently active toxic of any kind—bacterial toxin, poison, etc.—to induce excessive metabolism of its intrinsic cellular elements, and thus cause exhaustion or molecular metamorphosis of the latter.

" 9. The functional activity of the adrenals is decreased proportionally with that of the anterior pituitary body, whether the reduced activity of the latter be due to active or passive pathogenic factors."

It will thus be seen that the anterior pituitary body, instead of being, as generally supposed, the least important portion of the organism, assumes at once a function heretofore unrecognized,—that of regulating the amount of oxygen absorbed from the air by controlling the secretion of the suprarenal glands. The diminutive organ becomes, if this view be sustained, the most important organ of the body and the " governing centre of all oxidizing processes."

In relation to the posterior pituitary body the author says, " The posterior pituitary body is the general centre of the organism from which all the nervous energy transmitted by the bulbar centres arises."

The author apparently sums up his conclusions in the following paragraph:

" Briefly, our inquiry seems to us to have shown that the adrenal system is the source of the secretion which with the oxygen of the air forms the oxidizing substance of the blood-plasma. It has also revealed, we believe, the origin and mode of distribution of the bodies with which this oxygen directly or indirectly combines. . . . Finally, it has suggested that in addition to these agencies all leucocytes and, under certain circumstances, the plasma contain a protective agency, — trypsin, — which, with Metchnikoff's phagocytic cells, serves to destroy micro-organisms and convert their toxins and other albuminoid poisons into harmless products."

The author's views have been given principally in his own language, and the selections have been made in an effort to give his conclusions as briefly as possible. It is, however, recognized that this review imperfectly represents his laborious work. This covers so much ground that any attempt to give it adequate treament must result in failure.

The question, Will Dr. Sajous's deductions be accepted? cannot be answered here. Credit must be given for the original thought and the foundation which he has laid, broad and deep, for others to build upon in future investigations. This contribution to medical science must be regarded as among the most remarkable that the close of the nineteenth century and the early years of the twentieth have produced. Whoever undertakes to antagonize the positions and deductions taken must be prepared to show wherein the author is wrong, and that through exhaustive experimental procedures. In this way only can the truth or falsity of his conclusions be established.

It does not seem unreasonable to conclude that the two volumes will have a wide circulation. That the first volume deserves this is beyond question. It would be a valuable addition to any medical library if it had no other reason than that of the extensive literature quoted.

The first volume is presented in the satisfactory manner usual with the F. A. Davis Company.

ORTHODONTIA. A Text-Book for the Use of Students in Dental Colleges and a Hand-Book for Dental Practitioners. By J. N. MacDowell, D.D.S., Professor of Orthodontia in the College of Dentistry, University of Illinois, etc. Illustrated. E. H. Colegrove, 65 Randolph Street, Chicago, Ill.

There is no branch of dentistry that has received more attention than that of orthodontia, and yet there is, probably, no work that the average dentist is called upon to perform that is more distasteful to the operator than the regulating of teeth. To the artistic mind, coupled with mechanical skill, this work appeals as no other, with the result that there has been a multiplication of text-books on this subject, each having a distinct value, and from present indications the end is not yet.

This work of Dr. MacDowell is, in many respects, a very valuable addition to the list of books on this subject. It has been the aim of the author to "make it a most thorough and practical text-book on orthodontia for lectures, dental students, and practitioners." That he has succeeded in this must be admitted.

While Chapters I. and II., on "Comparative Dental Anatomy," have a certain value, they do not seem to be appropriate to a work of this character. It is difficult to see what relation exists between the teeth of vertebrates, fishes, reptiles, mammals, etc., and the irregularities of the human denture, and this is not made clear in the text.

The real work of the author does not begin until Chapter IV. is reached, when he takes up "Temporary Teeth, Anomalies, Eruption, Occlusion, etc. This is an excellent chapter, the deciduous teeth being too often omitted in books on orthodontia. This is naturally followed by the "Permanent Teeth: Eruption and Occlusion." "Anomalies of the Teeth classified" is the subject of Chapter VI. Passing on over the intervening chapters to Chapter IX., in which the author covers the various "regulating appliances" of a number of writers, including those of his own, the reader opens on Chapters X. and XI., on "Making Appliances, Wire, Tubing, Tools, Nuts, Taps," etc., and "Soldering Technique." These will appeal to the practical man. All, it is presumed, will agree with the author when he says, "The special requirements of these appliances should be simplicity, efficiency, stability, cleanliness, and inconspicuousness." These two chapters are of special value, giving the practitioner methods of producing appliances in the best and simplest manner. One not familiar with the work could not, it seems to the writer, fail to do it well if the very lucid description of how to prepare these is strictly followed.

The author does not favor "cutting away bone in front of a tooth." He says, "Occasionally there are cases favorable for assistance by this method, but they are few in number."

In Chapter XX. the author enters more at length into "Appliances and Methods of Treatment."

Chapter XXVIII., on "Retention," constitutes the last chapter but one of the book, and must be considered one of the most important, for without skill in the formation of retention appliances, coupled with judgment in placing them, the work of the orthodontist is valueless.

The book is most profusely and, upon the whole, very satisfactorily illustrated.

That the author has succeeded in presenting a very practical work must be admitted, and one that should be very helpful both to students and practitioners.

A Brief Manual of Prescription Writing in Latin or English. For the use of Physicians, Pharmacists, and Medical and Pharmacal Students. By M. L. Neff, A.M., M.D. F. A. Davis Company, Publishers, Philadelphia.

While this has been prepared for medical men and pharmacists, it is of equal value for dentists, especially dental students

The author says, "The notes here printed are the outgrowth of experience in teaching medical students, and are offered to a wider public in the hope that they will be of service to the physician and pharmacist."

To write a prescription properly some knowledge of Latin is requisite, and the author, recognizing this, devotes some thirty-four pages to this portion of the subject, although he "wishes to disclaim any attempt to teach the Latin language, as such." While this is true, this part of the book is made so clear that any one should be able to follow the author and succeed in acquiring sufficient knowledge of the language to write a prescription creditably.

The principal defect in the book seems to be in the altogether too limited space given the metric system. As this is coming more and more into use, it should have claimed a more generous share of attention.

The book is well worthy a place as a text-book in dental colleges, where something of the kind is very much needed.

